# EXCLUSION SPOTTER: APPLYING ADVANCES IN AI TO IDENTIFY AGEISM IN ONLINE JOB POSTING

**DOI:** 10.1093/geroni/igz038.2805

**Published:** 2019-11-08

**Authors:** Nicola Palmarini, Lee Martie, Mattie F Wasiak, Gaoyuan Zhang

**Affiliations:** 1 IBM Research, Cambridge, Massachusetts, United States; 2 Massachusetts Institute of Technology, Cambridge, Massachusetts, United States

## Abstract

The landscape in which employers and candidates interact is changing as more job adverts are pushed online. Employment platforms (e.g., Indeed and LinkedIn) are now among the primary mechanisms for job posting, job search, and initial negotiations. Through such job platforms, a single job advert can now reach millions of people around the world. This exposure of a job advert has obvious benefits for the employer, but this exposure also has the power to alienate and exclude large portions of society. In particular, the word choice of a single job advert can, perhaps unintentionally, exclude thousands of people by their personal traits (e.g., gender or race). Age is a particular trait that garners more attention as ageism is often cited in the literature as going overlooked, not understood, and generally escaping social awareness. To begin tackling this problem, with the purpose of supporting older adults and enabling their contribution to society, we applied advances in AI to create a tool, called Exclusion Spotter, that gives feedback to recruiters and employers on which words in their advert are possibly excluding people by age. We applied Exclusion Spotter to 3660 job adverts, clustered by 372 job titles. We found a significant difference (p=.02) in the number of age-related words for engineering related positions versus all other job titles. Among 47 engineering related titles we matched 47.37 age related words per title and 2.8 per advert. Among the other 325 titles we matched 24.37 age related words per title and 2.1 per advert.

